# Experimental solubility and modeling of Crizotinib (anti-cancer medication) in supercritical carbon dioxide

**DOI:** 10.1038/s41598-022-22366-y

**Published:** 2022-10-19

**Authors:** Gholamhossein Sodeifian, Chandrasekhar Garlapati, Armin Roshanghias

**Affiliations:** 1 Department of Chemical Engineering, Faculty of Engineering, University of Kashan, Kashan, 87317-53153 Iran; 2 Laboratory of Supercriritcal Fluids and Nanotechnology, University of Kashan, Kashan, 87317-53153 Iran; 3 Faculty of Engineering, Modeling and Simulation Centre, University of Kashan, Kashan, 87317-53153 Iran; 4 Department of Chemical Engineering, Puducherry Technological University, Puducherry, 605014 India

**Keywords:** Chemical engineering, Chemical engineering

## Abstract

Measurement of saturation solubility of drugs in a supercritical fluid is an important parameter for the implementation of supercritical technology in pharmaceutical industry. CO_2_ is the most sorted substance as a supercritical fluid since it has attractive properties like easily achievable critical temperature, moderate pressure. Cancer is increasingly affecting the mankind, a proper dosage while treating would help in minimizing the drug usage. The bioavailability of the drug is mainly influenced by the drug particle size. An appropriate technology is always useful in making suitable drug particles; thus, supercritical fluid technology (SFT) is considered as promising technique for the production of micro and nanoparticles. Since, particle production process through SFT needs solubility information, appropriate solubility information is necessary. In the present work, Crizotinib (anti-cancer drug) solubility in supercritical carbon dioxide (scCO_2_) is measured and reported, for the first time. The obtained solubilities are at temperatures 308, 318, 328,338 K and pressures 12, 15, 18, 21, 24 to 27 MPa. The measured solubilities are ranged in terms of mole fraction from (0.483 × 10^−5^ to 0.791 × 10^−5^) at 308 K, (0.315 × 10^−5^ to 0.958 × 10^−5^) at 318 K, (0.26 × 10^−5^ to 1.057 × 10^−5^) at 328 K, (0.156 × 10^−5^ to 1.219 × 10^−5^) at 338 K. The cross over region is observed at 14.5 MPa. To expand the application of the solubility data, few important solubility models and three cubic equations of sate (cubic EoS) models along with Kwak and Mansoori mixing rules are investigated. Sublimation and salvation enthalpies of Crizotinib dissolution in scCO_2_ are calculated.

## Introduction

Traditionally, pharmaceutical particle production processes use several organic solvents for processing their products. Quite often, remnant of solvents causes serious pollution and sometimes reactions with pharmaceutical products and result in unnecessary byproducts. These issues are effectively handled using supercritical fluid technologies (SFTs). Although, there is a scope of using several substances as supercritical fluids, CO_2_ as supercritical fluid has gained importance for the last three decades. Commonly, all supercritical fluids (SCFs) have gas like diffusivities and liquid like densities makes them attractive for extraction processes. When CO_2_ pressure and temperature conditions are maintained above 7.39 MPa and 304.15 K, it will act as supercritical fluid and it is commonly abbreviated as supercritical carbon dioxide (scCO_2_)^1–4^. Due to these features, it is used as a solvent in various process applications^1–8^.Some of the major applications of scCO_2_ in process industry are pharmaceutical particle size design, food processing, textile dyeing and extraction^1–4^.Solubility information is necessary for the proper implementation of SFTs for particle size design process. The task of measuring solubility of important anti-cancer drugs in scCO_2_ is taken in this work. In recent literature, solubilities of several anti-cancer drugs in scCO_2_ are readily available^8–26^, but the Crizotinib solubility in scCO_2_ is not reported, therefore in this work for the first time its solubility is measured and reported. Crizotinib is used in the treatment of some kind of non-small cell lung cancer (NSCLC) that spreads to nearby tissues or to other parts of the body^27^.Crizotinib helps to stop the growth of tumor cells by blocking the anaplastic lymphoma kinase (ALK) protein from working; it is also termed as fusion mutation. Appropriate dosage is attained with proper drug particle size and it is very critical for the treatment of cancer. Thus, the information obtained this study is useful in preparing various size drug particles using scCO_2_. For practical purpose obtaining solubilities at various conditions are cumbersome hence model identification for the solubility is essential^28^. The data developed in this work are tested with few important solubility models and three cubic equation of state models (cubic EoS) along with Kwak and Mansoori mixing rules^29^.

The purpose of this study is in two levels. In the first level, Crizotinib solubility in scCO_2_ is determined and in the second, data obtained are correlated with existing solubility models and with three Cubic EoS models along with Kwak and Mansoori mixing rules.

## Experimental

### Materials

Crizotinib is supplied by Amin Pharma company (CAS Number: 877399-52-5, mass purity > 99%). CO_2_ (CAS Number: 124-38-9, mass purity > 99.9%) is purchased from Fadak company, Kashan (Iran). Dimethyl sulfoxide (DMSO, CAS Number: 67-68-5, mass purity > 99%) is procured from Sigma Aldrich company. Table 1 indicates the information about the chemicals utilized in this work.

**Table 1:** Molecular structure and physicochemical properties of used materials.

Compound	Formula	Structure	M_W_ (g/mol)	λ_max_(nm)	CAS number	Minimum purity (mass) (%)
Crizotinib	C_21_H_22_Cl_2_FN_5_O		450.3	270	877399-52-5	99
Carbon dioxide	CO_2_		44.01		124-38-9	99.99
DMSO	C_2_H_6_OS		78.13		67-68-5	99

### Experiment details

**Figure 1:**
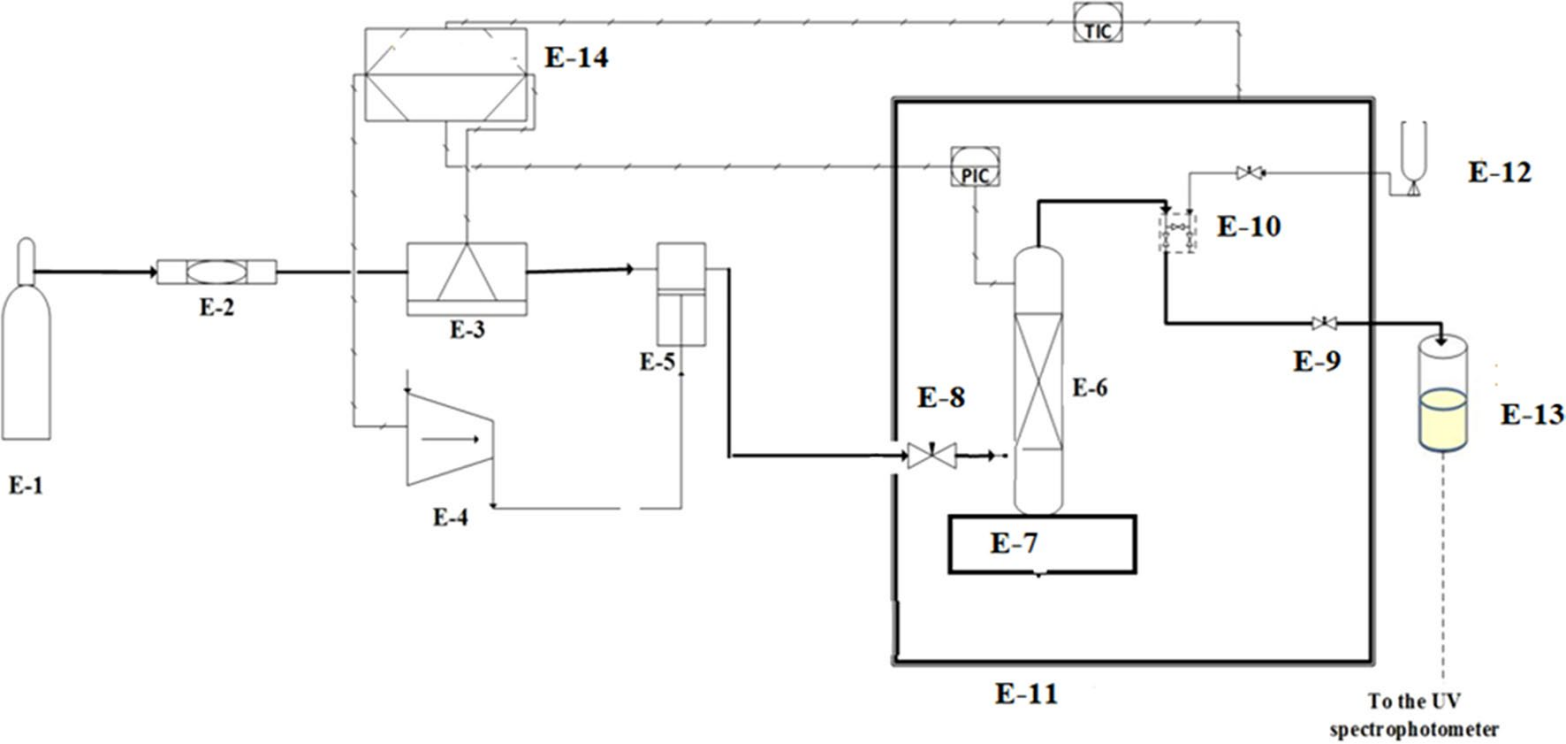
Experimental setup for solubility measurement, E1—CO_2_ cylinder; E-2—Filter; E-3—Refrigerator unit; E-4—Air compressor; E-5—High pressure pump; E-6—Equilibrium cell; E-7—Magnetic stirrer; E-8—Needle valve; E-9—Back-pressure valve; E-10 Six-port, two position valve; E-11—Oven; E-12—Syringe; E13—Collection vial; E-14—Control panel.

The experimental setup utilized in this study is shown in Fig. 1. The details about the solubility apparatus and experimental procedure are presented in our earlier studies (Fig. 1)^30–42^. However, a brief description is presented in this section. The method of solubility measurement utilized is classified as an isobaric-isothermal method^43^. All measurements were made with high precision while controlling temperatures and pressures within ± 0.1 K and ± 0.1 MPa, respectively. Solubility measurements were performed at least in triplicate for each data point. For each measurement, 1 g of Crizotinib drug was used. In line with our previous works, for this compound, the observed equilibrium was within 60 min. After equilibrium, 600 µL saturated scCO_2_ sample was collected via 2-status 6-way port valve in a DMSO preloaded vial. After discharging 600 µL saturated scCO_2_, the port valve was washed with 1 ml DMSO. Thus, the total saturation solution was 5 ml. Drug solubility in terms of mole fraction is calculated with the following formula:

1 $$y_{2} = \frac{{n_{drug} }}{{n_{drug} + n_{{CO_{2} }} }}$$

where $n_{{{\text{drug}}}}$ is number of moles of the drug, and $n_{{{\text{CO}}_{{2}} }}$ is number of moles of CO_2_ in the sampling loop.

Furthermore, the above quantities are given as:

2 $$n_{{{\text{drug}}}} = \frac{{C_{s} \cdot V_{s} }}{{M_{s} }}$$

3 $$n_{{{\text{CO}}_{{2}} }} = \frac{{V_{1} \cdot \rho }}{{M_{{{\text{CO}}_{{2}} }} }}$$

where $C_{{\text{s}}}$ is the drug concentration in saturated sample vial in g/L. The volume of the sampling loop and vial collection are V_1_(L) = 600 $\times$ 10^−6^ m^3^ and V_s_(L) = 5 $\times$ 10^−3^ m^3^, respectively. The $M_{s}$ and $M_{{CO_{2} }}$ are the molecular weights of the drug and CO_2_, respectively. Solubility is also described as

4 $$S = \frac{{C_{S} V_{s} }}{{V_{1} }}$$

The relation between S and $y_{2}$ is

5 $$S = \frac{{\rho M_{s} }}{{M_{{CO_{2} }} }}\frac{{y_{2} }}{{1 - y_{2} }}$$

The Crizotinib’s solubility was determined using a UV–Visible spectrophotometer (Model UNICO-4802) and DMSO solvent was used for the measurement of its solubility. Drug samples were analyzed at wave length of 270 nm.

## Modeling

The Crizotinib’s solubility data measured in this study is correlated by four standard solubility models namely Chrastil, Modefied Chrastil, Mendez-Santiago and Teja and Bartle et al. Moreover, three cubic EoS models along with Kwak and Mansoori mixing rules are explored for the solubility data correlation. More details about the models considered in this work are presented in the following subsections^6^.

### Standard solubility models

#### Chrastil model^44^

Chrastil model represents by Eq. (6) that relates solubility mole fraction in terms of solvent density, association number and temperature

6 $$c_{2} = \rho_{1}^{\kappa } \exp \left( {A_{1} + \frac{{B_{1} }}{T}} \right)$$

where model constants are $\kappa ,A_{1} \;{\text{and}}\;B_{1}$.

Equation (6) is alternatively represented as^45^

7 $$y_{2} = \frac{{\left( {\rho_{1} } \right)^{\kappa - 1} \exp \left( {A_{1} + \frac{{B_{1} }}{T}} \right)}}{{\left[ {1 + \left( {\rho_{1} } \right)^{\kappa - 1} \exp \left( {A_{1} + \frac{{B_{1} }}{T}} \right)} \right]}}$$

where $\kappa ,A_{1} \;{\text{and}}\;B_{1}$ are the model parameters.

#### Modified Chrastil model^46^

Dimensionally corrected Charstil model is also known as modified Charstil model and is described in terms of solvent density, association number and temperature as

8 $$y_{2} = \left( {\frac{{RT\rho_{1} }}{{M_{ScF} f^{ \bullet } }}} \right)^{{\kappa^{\prime} - 1}} \exp \left( {A_{2} + \frac{{B_{2} }}{T}} \right)$$

where $\kappa^{\prime},A_{2} \;{\text{and}}\;B_{2}$ are the model parameters.

#### Méndez-Santiago and Teja (MT) model^47^

Measured data self-consistency is checked with this model and represents by Eq. (9)

9 $$T\ln \left( {y_{2} P} \right) = A_{3} + B_{3} \rho_{1} + C_{3} T$$

where *A*_3_ and *C*_3_ are the model parameters.

#### Bartle et al., model^48^

Sublimation enthalpy of the dissolved solids in SCFs is measured with this model and stated as

10 $$\ln \left( {\frac{{y_{2} P}}{{P_{ref} }}} \right) = A_{4} + \frac{{B_{4} }}{T} + C_{4} \left( {\rho_{1} - \rho_{ref} } \right)$$

where *A*_4_*, B*_4_ and *C*_4_ are the model parameters. From parameter *B*_4_, sublimation enthalpy is estimated ($\Delta_{sub} H = - B_{4} R$).

### Equation of sate (EoS) modeling

There are several EoS models among them Redlich-Kwong (RK), Soave–Redlich–Kwong (SRK) and Peng-Robinson (PR) EoSs are commonly used in correlating solubility of solid compounds in scCO_2_.All these models for correlation requires adjustable interaction parameters and they are found to be temperature dependent^49,50^. In the year 1986 Kwak and Mansoori developed a new concept in the spirit of van der Waals mixing rules (VdWmrs), which resulted in temperature independent interaction parameters. They have demonstrated the correlating ability of RK and PR EoSs^29^. However, SRK EoS has not been explored, therefore in this work, the SRK EoS along with Kwak and Mansoori mixing rules are explored and finally two forms of solubility models for SRK EoS model is proposed. More details about the EoS modeling are presented in the following subsections.

#### RK EoS with Kwak and Mansoori mixing rules^29^

RK EoS in terms of compressibility factor (Z) is given by

11 $$Z = \frac{v}{v - b} - \frac{a}{{RT^{1.5} \left( {v + b} \right)}} \,$$

VdWmrs for RK EoS are expressed as

12 $$a = \frac{{\left( {\sum\limits_{i}^{n} {\sum\limits_{j}^{n} {x_{i} x_{j} a_{ij}^{{{\raise0.7ex\hbox{$2$} \!\mathord{\left/ {\vphantom {2 3}}\right.\kern-\nulldelimiterspace} \!\lower0.7ex\hbox{$3$}}}} b_{ij}^{{{\raise0.7ex\hbox{$1$} \!\mathord{\left/ {\vphantom {1 3}}\right.\kern-\nulldelimiterspace} \!\lower0.7ex\hbox{$3$}}}} } } } \right)^{{{\raise0.7ex\hbox{$3$} \!\mathord{\left/ {\vphantom {3 2}}\right.\kern-\nulldelimiterspace} \!\lower0.7ex\hbox{$2$}}}} }}{{\left( {\sum\limits_{i}^{n} {\sum\limits_{j}^{n} {x_{i} x_{j} b_{ij}^{{{\raise0.7ex\hbox{$1$} \!\mathord{\left/ {\vphantom {1 2}}\right.\kern-\nulldelimiterspace} \!\lower0.7ex\hbox{$2$}}}} } } } \right)^{{{\raise0.7ex\hbox{$1$} \!\mathord{\left/ {\vphantom {1 2}}\right.\kern-\nulldelimiterspace} \!\lower0.7ex\hbox{$2$}}}} }}$$

13 $$b = \sum\limits_{i}^{n} {\sum\limits_{j}^{n} {x_{i} x_{j} b_{ij} } }$$

14 $$a_{ij} = \left( {1 - k_{ij} } \right)\sqrt {a_{ii} a_{jj} }$$

15 $$b_{ij} = \left( {1 - l_{ij} } \right)\frac{{\left( {b_{ii}^{{^{{{\raise0.7ex\hbox{$1$} \!\mathord{\left/ {\vphantom {1 3}}\right.\kern-\nulldelimiterspace} \!\lower0.7ex\hbox{$3$}}}} }} + b_{jj}^{{{\raise0.7ex\hbox{$1$} \!\mathord{\left/ {\vphantom {1 3}}\right.\kern-\nulldelimiterspace} \!\lower0.7ex\hbox{$3$}}}} } \right)^{3} }}{8}$$

Equations (12) to (15) combined with Eq. (11), will constitute the RK EoS for mixtures, consistent with the statistical-mechanical basis of the VdW mixing rules.

#### PR EoS with Kwak and Mansoori mixing rules^29,49,50^

PR EoS in terms of compressibility factor (Z) is given by

16 $$Z = \frac{v}{v - b} - \frac{{{\raise0.7ex\hbox{$a$} \!\mathord{\left/ {\vphantom {a {RT}}}\right.\kern-\nulldelimiterspace} \!\lower0.7ex\hbox{${RT}$}} + c - 2\sqrt {\frac{ac}{{RT}}} }}{{\left( {v + b} \right) + \left( {{\raise0.7ex\hbox{$b$} \!\mathord{\left/ {\vphantom {b v}}\right.\kern-\nulldelimiterspace} \!\lower0.7ex\hbox{$v$}}} \right)\left( {v - b} \right)}} \,$$

where

$$a{\kern 1pt} \left( T \right) = 0.45724\frac{{R^{2} T_{c}^{2} }}{{P_{c} }}\alpha \left( {T_{r} ,\omega } \right) \,$$

$$\alpha \left( {T_{r} ,\omega } \right) = \left[ {1 + \left( {0.37464 + 1.5422\omega - 0.26992\omega^{2} \left( {1 - \sqrt {T_{r} } } \right)} \right)} \right]^{2}$$

$$b = 0.07780\frac{{RT_{c} }}{{P_{c} }}$$

$$a = a \, (T_{c} ) \, \left( {1 + m} \right)^{2} \, and \, c = {\raise0.7ex\hbox{${a \, (T_{c} ) \, m^{2} \, }$} \!\mathord{\left/ {\vphantom {{a \, (T_{c} ) \, m^{2} \, } {RT_{c} }}}\right.\kern-\nulldelimiterspace} \!\lower0.7ex\hbox{${RT_{c} }$}}$$

Equation (16) provides three independent constants in terms of *a*, *b* and *c*. Now following the guidelines given by Kwak and Mansoori mixing rules result in the following expressions for *a*, *b* and *c*

17 $$a = \sum\limits_{i}^{n} {\sum\limits_{j}^{n} {x_{i} } } x_{j} a_{ij}$$

18 $$b = \sum\limits_{i}^{n} {\sum\limits_{j}^{n} {x_{i} } } x_{j} b_{ij}$$

19 $$c = \sum\limits_{i}^{n} {\sum\limits_{j}^{n} {x_{i} } } x_{j} c_{ij}$$

With the following interaction parameters

20 $${\text{a}}_{{{\text{ij}}}} = \left( {1 - k_{ij} } \right)\sqrt {a_{ii} } a_{jj}$$

21 $${\text{b}}_{{{\text{ij}}}} = \left( {{\text{1 - l}}_{{{\text{ij}}}} } \right) \, \left( {\frac{{{\text{b}}_{{{\text{ii}}}}^{{{\raise0.7ex\hbox{${1}$} \!\mathord{\left/ {\vphantom {{1} {3}}}\right.\kern-\nulldelimiterspace} \!\lower0.7ex\hbox{${3}$}}}} + b_{jj}^{{{\raise0.7ex\hbox{$1$} \!\mathord{\left/ {\vphantom {1 3}}\right.\kern-\nulldelimiterspace} \!\lower0.7ex\hbox{$3$}}}} }}{2}} \right)^{{3}}$$

22 $${\text{ c}}_{{{\text{ij}}}} = \left( {{\text{1 - m}}_{{{\text{ij}}}} } \right) \, \left( {\frac{{{\text{c}}_{{{\text{ii}}}}^{{{\raise0.7ex\hbox{${1}$} \!\mathord{\left/ {\vphantom {{1} {3}}}\right.\kern-\nulldelimiterspace} \!\lower0.7ex\hbox{${3}$}}}} + c_{jj}^{{{\raise0.7ex\hbox{$1$} \!\mathord{\left/ {\vphantom {1 3}}\right.\kern-\nulldelimiterspace} \!\lower0.7ex\hbox{$3$}}}} }}{2}} \right)^{{3}}$$

Equations (17) to (22) combined with Eq. (16), will constitute the PR EoS for mixtures, consistent with the statistical-mechanical basis of the VdW mixing rules.

#### SRK EoS with Kwak and Mansoori mixing rules^38^

In the following, SRK EoS is given by^51^

23 $$P = \frac{RT}{{V - b}} - \frac{a\left( T \right)}{{V\left( {V + b} \right)}} \,$$

where *V* is the molar volume and other parameters have usual meanings. The pure component parameter *a*, which is a function of temperature and *b*, which is a constant and they are obtained from the following relations.

24 $$a\left( T \right) = 0.42748\frac{{R^{2} T_{c}^{2} }}{{P_{c} }}\alpha \left( T \right) \,$$

25 $$b = 0.08664\frac{{RT_{c} }}{{P_{c} }} \,$$

26 $$\alpha \left( T \right) = \left( {1 + m\left( {1 - \sqrt {\frac{T}{{T_{c} }}} } \right)} \right)^{2} \,$$

where *m* is a constant given by

27 $$m = 0.48 + 1.574\omega - 0.176\omega^{2}$$

where ‘ω’ is the acentric factor. In 1993, Soave proposed a new α(T) function for heavy hydrocarbons to be used with SRK EoS^52^

28 $$\alpha \left( T \right) = 1 + m\left( {1 - \frac{T}{{T_{c} }}} \right) + n\left( {1 - \sqrt {\frac{T}{{T_{c} }}} } \right)^{2}$$

where

29 $$m = 0.484 + 1.515\omega - 0.044\omega^{2} \quad$$

30 $$and\quad n = 2.756m - 0.7 \,$$

In order to separate thermodynamic variables from constants of the SRK EoS, we have adopted the following two ways.

When Eqs. (26), (27) and (23) are combined to get the following form for SRK EoS in terms of compressibility factor (Z)

31 $$Z = \frac{v}{v - b} - \frac{{{\raise0.7ex\hbox{$a$} \!\mathord{\left/ {\vphantom {a {RT}}}\right.\kern-\nulldelimiterspace} \!\lower0.7ex\hbox{${RT}$}} + c - 2\sqrt {\frac{ac}{{RT}}} }}{v + b} \,$$

where

$$a = a \, (T_{c} ) \, \left( {1 + m} \right)^{2} {\text{ and c}} = {\raise0.7ex\hbox{${{\text{a (T}}_{{\text{c}}} {\text{) m}}^{{2}} \, }$} \!\mathord{\left/ {\vphantom {{{\text{a (T}}_{{\text{c}}} {\text{) m}}^{{2}} \, } {RT_{c} }}}\right.\kern-\nulldelimiterspace} \!\lower0.7ex\hbox{${RT_{c} }$}}$$

This form of the SRK EoS suggests three independent constants namely *a*, *b* and c. Now following the guidelines given by Kwak and Mansoori mixing rules result in following expressions for *a*, *b* and *c*

32 $$a = \sum\limits_{i}^{n} {\sum\limits_{j}^{n} {x_{i} } } x_{j} a_{ij}$$

33 $$b = \sum\limits_{i}^{n} {\sum\limits_{j}^{n} {x_{i} } } x_{j} b_{ij}$$

34 $$c = \sum\limits_{i}^{n} {\sum\limits_{j}^{n} {x_{i} } } x_{j} c_{ij}$$

With the following interaction parameters:

35 $${\text{a}}_{{{\text{ij}}}} = \left( {1 - k_{ij} } \right)\sqrt {a_{ii} } a_{jj}$$

36 $${\text{ b}}_{{{\text{ij}}}} = \left( {{\text{1 - l}}_{{{\text{ij}}}} } \right) \, \left( {\frac{{{\text{b}}_{{{\text{ii}}}}^{{{\raise0.7ex\hbox{${1}$} \!\mathord{\left/ {\vphantom {{1} {3}}}\right.\kern-\nulldelimiterspace} \!\lower0.7ex\hbox{${3}$}}}} + b_{jj}^{{{\raise0.7ex\hbox{$1$} \!\mathord{\left/ {\vphantom {1 3}}\right.\kern-\nulldelimiterspace} \!\lower0.7ex\hbox{$3$}}}} }}{2}} \right)^{{3}} \,$$

37 $${\text{ c}}_{{{\text{ij}}}} = \left( {{\text{1 - m}}_{{{\text{ij}}}} } \right) \, \left( {\frac{{{\text{c}}_{{{\text{ii}}}}^{{{\raise0.7ex\hbox{${1}$} \!\mathord{\left/ {\vphantom {{1} {3}}}\right.\kern-\nulldelimiterspace} \!\lower0.7ex\hbox{${3}$}}}} + c_{jj}^{{{\raise0.7ex\hbox{$1$} \!\mathord{\left/ {\vphantom {1 3}}\right.\kern-\nulldelimiterspace} \!\lower0.7ex\hbox{$3$}}}} }}{2}} \right)^{{3}} \,$$

Equations (32) to (37) combined with Eq. (31), will constitute the SRK EoS for mixtures, consistent with the statistical-mechanical basis of the VdW mixing rules.

When Eqs. (28), (29), (30) and (23) are combined to get the following form.

38 $$Z = \frac{v}{v - b} - \frac{{{\raise0.7ex\hbox{$a$} \!\mathord{\left/ {\vphantom {a {RT}}}\right.\kern-\nulldelimiterspace} \!\lower0.7ex\hbox{${RT}$}} + c - {\raise0.7ex\hbox{$d$} \!\mathord{\left/ {\vphantom {d {\sqrt T }}}\right.\kern-\nulldelimiterspace} \!\lower0.7ex\hbox{${\sqrt T }$}}}}{v + b} \,$$

where

$$a = a \, (T_{c} ) \, \left( {1 + m + n} \right){\text{ , c}} = \frac{{a \, (T_{c} ) \, \left( {n - m} \right)}}{{RT_{c} }}{\text{ and d}} = {\raise0.7ex\hbox{${{\text{2n a (T}}_{{\text{c}}} )}$} \!\mathord{\left/ {\vphantom {{{\text{2n a (T}}_{{\text{c}}} )} {R\sqrt {T_{c} } }}}\right.\kern-\nulldelimiterspace} \!\lower0.7ex\hbox{${R\sqrt {T_{c} } }$}}$$

This form of the SRK EoS suggests that four independent constants exist in the EoS namely *a*, *b*, *c* and *d*. Now following the guidelines given by Kwak and Mansoori mixing rules results in following expressions for *a*, *b*, *c* and *d*

39 $$a = \sum\limits_{i}^{n} {\sum\limits_{j}^{n} {x_{i} } } x_{j} a_{ij}$$

40 $$b = \sum\limits_{i}^{n} {\sum\limits_{j}^{n} {x_{i} } } x_{j} b_{ij}$$

41 $$c = \sum\limits_{i}^{n} {\sum\limits_{j}^{n} {x_{i} } } x_{j} c_{ij}$$

42 $$d = \sum\limits_{i}^{n} {\sum\limits_{j}^{n} {x_{i} } } x_{j} d_{ij}$$

With the following interaction parameters:

43 $${\text{a}}_{{{\text{ij}}}} = \left( {1 - k_{ij} } \right)\sqrt {a_{ii} } a_{jj}$$

44 $${\text{ b}}_{{{\text{ij}}}} = \left( {{\text{1 - l}}_{{{\text{ij}}}} } \right) \, \left( {\frac{{{\text{b}}_{{{\text{ii}}}}^{{{\raise0.7ex\hbox{${1}$} \!\mathord{\left/ {\vphantom {{1} {3}}}\right.\kern-\nulldelimiterspace} \!\lower0.7ex\hbox{${3}$}}}} + b_{jj}^{{{\raise0.7ex\hbox{$1$} \!\mathord{\left/ {\vphantom {1 3}}\right.\kern-\nulldelimiterspace} \!\lower0.7ex\hbox{$3$}}}} }}{2}} \right)^{{3}}$$

45 $${\text{ c}}_{{{\text{ij}}}} = \left( {{\text{1 - m}}_{{{\text{ij}}}} } \right) \, \left( {\frac{{{\text{c}}_{{{\text{ii}}}}^{{{\raise0.7ex\hbox{${1}$} \!\mathord{\left/ {\vphantom {{1} {3}}}\right.\kern-\nulldelimiterspace} \!\lower0.7ex\hbox{${3}$}}}} + c_{jj}^{{{\raise0.7ex\hbox{$1$} \!\mathord{\left/ {\vphantom {1 3}}\right.\kern-\nulldelimiterspace} \!\lower0.7ex\hbox{$3$}}}} }}{2}} \right)^{{3}} \,$$

46 $${\text{ d}}_{{{\text{ij}}}} = \left( {{\text{1 - n}}_{{{\text{ij}}}} } \right) \, \left( {\frac{{{\text{d}}_{{{\text{ii}}}}^{{{\raise0.7ex\hbox{${1}$} \!\mathord{\left/ {\vphantom {{1} {3}}}\right.\kern-\nulldelimiterspace} \!\lower0.7ex\hbox{${3}$}}}} + d_{jj}^{{{\raise0.7ex\hbox{$1$} \!\mathord{\left/ {\vphantom {1 3}}\right.\kern-\nulldelimiterspace} \!\lower0.7ex\hbox{$3$}}}} }}{2}} \right)^{{3}}$$

Equations (39) to (46) combined with Eq. (38), will constitute the SRK EoS for mixtures, consistent with the statistical-mechanical basis of the VdW mixing rules.

### EoS model for the solubility of solids in scCO_2_

The mole fraction of dissolved solid drug *i* (solute) in Solvent scCO_2_ is expressed as^53^

47 $${\text{y}}_{{\text{i}}} = \frac{{P_{i}^{S} \hat{\phi }_{i}^{S} }}{{P\hat{\phi }_{i}^{{ScCO_{2} }} }}\exp \left[ {\frac{{\left( {P - P_{i}^{S} } \right)v_{S} }}{RT}} \right] \,$$

where $P_{i}^{s}$ is the sublimation pressure and other parameters have usual meanings. The saturation fugacity coefficient ($\hat{\phi }_{i}^{s}$) is assumed to be unity. The required expression for the solid solute fugacity coefficient in the ScCO_2_($\left( {\hat{\phi }_{i}^{{ScCO_{2} }} } \right)$) is calculated using three cubic EoS along with Kwak and Mansoori mixing rules. They are obtained from the following basic thermodynamic relation^54^

48 $$\ln \left( {\hat{\varphi }_{i}^{{ScCO_{2} }} } \right) = \frac{1}{RT}\int\limits_{v}^{\infty } {\left[ {\left( {\frac{\partial P}{{\partial N_{i} }}} \right)_{{T,V,N_{j} }} - \frac{RT}{v}} \right]} dv - \ln Z$$

Equations (49) to (52) represent the fugacity coefficients expressions used in this study;

For RK EoS

49 $$\begin{gathered} \ln \left( {\hat{\varphi }_{i}^{{ScCO_{2} }} } \right) = \ln \left( {\frac{v}{v - b}} \right) + \left( {\frac{{2\sum {x_{j} b_{ij} - b} }}{v - b}} \right) - \ln \left( Z \right) + \hfill \\ \left( {\frac{{a\left( {2\sum {x_{j} b_{ij} - b} } \right)}}{{b^{2} RT^{{{\raise0.7ex\hbox{$3$} \!\mathord{\left/ {\vphantom {3 2}}\right.\kern-\nulldelimiterspace} \!\lower0.7ex\hbox{$2$}}}} }}} \right)\left[ {\ln \left( {\frac{v + b}{v}} \right) - \frac{b}{v + b}} \right]\left( {3\alpha^{{{\raise0.7ex\hbox{$1$} \!\mathord{\left/ {\vphantom {1 2}}\right.\kern-\nulldelimiterspace} \!\lower0.7ex\hbox{$2$}}}} {{\left( {\sum {x_{j} a_{ij}^{{{\raise0.7ex\hbox{$2$} \!\mathord{\left/ {\vphantom {2 3}}\right.\kern-\nulldelimiterspace} \!\lower0.7ex\hbox{$3$}}}} b_{ij}^{{{\raise0.7ex\hbox{$1$} \!\mathord{\left/ {\vphantom {1 3}}\right.\kern-\nulldelimiterspace} \!\lower0.7ex\hbox{$3$}}}} } } \right)} \mathord{\left/ {\vphantom {{\left( {\sum {x_{j} a_{ij}^{{{\raise0.7ex\hbox{$2$} \!\mathord{\left/ {\vphantom {2 3}}\right.\kern-\nulldelimiterspace} \!\lower0.7ex\hbox{$3$}}}} b_{ij}^{{{\raise0.7ex\hbox{$1$} \!\mathord{\left/ {\vphantom {1 3}}\right.\kern-\nulldelimiterspace} \!\lower0.7ex\hbox{$3$}}}} } } \right)} {b^{{{\raise0.7ex\hbox{$1$} \!\mathord{\left/ {\vphantom {1 2}}\right.\kern-\nulldelimiterspace} \!\lower0.7ex\hbox{$2$}}}} }}} \right. \kern-\nulldelimiterspace} {b^{{{\raise0.7ex\hbox{$1$} \!\mathord{\left/ {\vphantom {1 2}}\right.\kern-\nulldelimiterspace} \!\lower0.7ex\hbox{$2$}}}} }} - \alpha^{{{\raise0.7ex\hbox{$2$} \!\mathord{\left/ {\vphantom {2 3}}\right.\kern-\nulldelimiterspace} \!\lower0.7ex\hbox{$3$}}}} \left( {{{\sum {x_{j} b_{ij} } } \mathord{\left/ {\vphantom {{\sum {x_{j} b_{ij} } } {b^{{{\raise0.7ex\hbox{$3$} \!\mathord{\left/ {\vphantom {3 2}}\right.\kern-\nulldelimiterspace} \!\lower0.7ex\hbox{$2$}}}} }}} \right. \kern-\nulldelimiterspace} {b^{{{\raise0.7ex\hbox{$3$} \!\mathord{\left/ {\vphantom {3 2}}\right.\kern-\nulldelimiterspace} \!\lower0.7ex\hbox{$2$}}}} }}} \right)} \right)/bRT^{{{\raise0.7ex\hbox{$3$} \!\mathord{\left/ {\vphantom {3 2}}\right.\kern-\nulldelimiterspace} \!\lower0.7ex\hbox{$2$}}}} \hfill \\ \end{gathered}$$

For PR EoS

50 $${\text{ln}}\left( {\hat{\phi }_{{\text{i}}}^{ScF} } \right) = \left( {\frac{{2\hat{B}}}{b} - 1} \right) \, \left( {\text{Z - 1}} \right){\text{ - ln}}\left[ {{\text{Z}}\left( {{1 - }\frac{b}{v}} \right)} \right] \, + \left[ {\frac{\Delta }{\sqrt 2 RTb}} \right] \, \times {\text{ln}}\left[ {\frac{{{1} + \left( {{1} + \sqrt {2} } \right)\frac{b}{v}}}{{{1} + \left( {{1 - }\sqrt {2} } \right)\frac{b}{v}}}} \right] \,$$

where

$$\begin{gathered} \Delta = \left[ {\frac{E}{2} - \frac{{E\hat{B}}}{b} + \hat{A}\left( {1 - \sqrt {{\raise0.7ex\hbox{${RTc}$} \!\mathord{\left/ {\vphantom {{RTc} a}}\right.\kern-\nulldelimiterspace} \!\lower0.7ex\hbox{$a$}}} } \right) + \hat{C}\left( {RT - \sqrt {{\raise0.7ex\hbox{${RTa}$} \!\mathord{\left/ {\vphantom {{RTa} c}}\right.\kern-\nulldelimiterspace} \!\lower0.7ex\hbox{$c$}}} } \right)} \right] \hfill \\ E = a + cRT - 2\sqrt {acRT} ;\hat{A} = \sum {x_{i} } a_{ij} ;\hat{B} = \sum {x_{i} } b_{ij} {\text{and }}\hat{C} = 2\sum {x_{i} } c_{ij} \hfill \\ \end{gathered}$$

For SRK EoS

When three parameters are considered

51 $$\ln \left( {\hat{\phi }_{i}^{ScF} } \right) = \frac{{\hat{b}}}{b} \, \left( {Z - 1} \right) \, - \ln \left[ {Z\left( {1 - \frac{b}{v}} \right)} \right] \, + \left[ {\frac{{E\hat{b}}}{{b^{2} RT}} - \frac{{\hat{E}}}{bRT}} \right] \, \ln \left( {1 + \frac{b}{v}} \right)$$

where

$$\begin{gathered} E = a + cRT - 2\sqrt {acRT} \hfill \\ \hat{E} = \frac{1}{N}\frac{{\partial \left( {N^{2} E} \right)}}{{\partial N_{i} }} = \hat{a} \, \left( {1 - \sqrt {{\raise0.7ex\hbox{${RTc}$} \!\mathord{\left/ {\vphantom {{RTc} a}}\right.\kern-\nulldelimiterspace} \!\lower0.7ex\hbox{$a$}}} } \right) + \hat{c}\left( {RT - \sqrt {{\raise0.7ex\hbox{${RTa}$} \!\mathord{\left/ {\vphantom {{RTa} c}}\right.\kern-\nulldelimiterspace} \!\lower0.7ex\hbox{$c$}}} } \right) \hfill \\ \hat{a} = 2\sum {x_{i} } a_{ij} ,\,\,\hat{b} = 2\sum {x_{i} } b_{ij} - b\;{\text{and}}\,\hat{c} = 2\sum {x_{i} } c_{ij} \hfill \\ \end{gathered}$$

When four parameters are considered

52 $$\ln \left( {\hat{\phi }_{i}^{ScF} } \right) = \frac{{\hat{b}}}{b} \, \left( {Z - 1} \right) \, - \ln \left[ {Z\left( {1 - \frac{b}{v}} \right)} \right] \, + \left[ {\frac{{E\hat{b}}}{{b^{2} RT}} - \frac{{\hat{E}}}{bRT}} \right] \, \ln \left( {1 + \frac{b}{v}} \right)$$

where

$$\begin{gathered} E = a + cRT - dR\sqrt T \hfill \\ \hat{E} = \frac{1}{N}\frac{{\partial \left( {N^{2} E} \right)}}{{\partial N_{i} }} = \hat{a} + RT\hat{c} - R\sqrt T \hat{d} \hfill \\ \hat{a} = 2\sum {x_{i} } a_{ij} { , }\quad \hat{b} = {2}\sum {x_{i} } b_{ij} - b{ , }\quad \hat{c} = 2\sum {x_{i} } c_{ij} {\text{ and }}\hat{d} = 2\sum {x_{i} } d_{ij} \hfill \\ \end{gathered}$$

For the data regression, fminsearch (MATLAB 2019) algorithm has been used. Solubility models are regressed with the following objective function^55^

53 $$OF = \sum\limits_{i = 1}^{N} {\frac{{\left| {y_{2i}^{\exp } - y_{2i}^{calc} } \right|}}{{y_{2i}^{\exp } }}}$$

Final results are indicated in terms of an average absolute relative deviation percentage (AARD %).

54 $${\text{AARD}}\left( {\text{\% }} \right) = \left( {{\raise0.7ex\hbox{${100}$} \!\mathord{\left/ {\vphantom {{100} {N_{i} }}}\right.\kern-\nulldelimiterspace} \!\lower0.7ex\hbox{${N_{i} }$}}} \right)\sum\limits_{i = 1}^{N} {\frac{{\left| {y_{2i}^{\exp } - y_{2i}^{cal} } \right|}}{{y_{2i}^{\exp } }}} .$$

## Results and discussion

**Figure 2:**
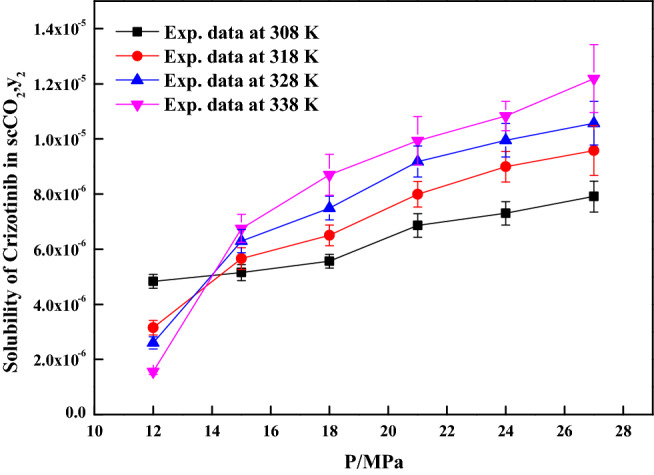
Solubility isotherms of Crizotinib in scCO_2_.

**Figure 3:**
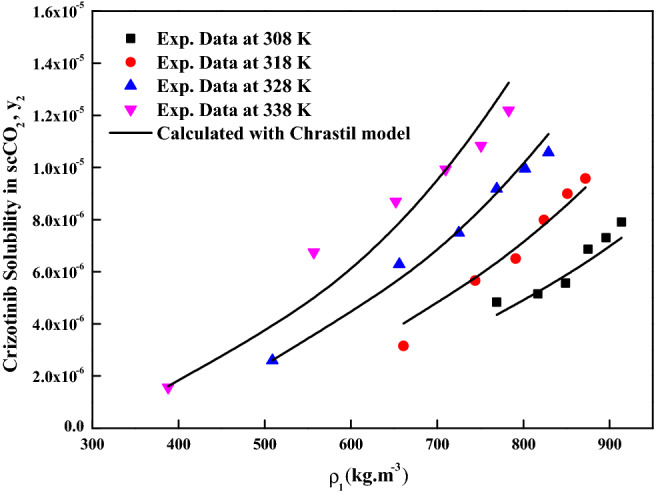
Crizotinib solubility in scCO_2_ versus ρ_1_. Symbols are experimental data points. Solid lines are calculated solubilities with Chrastil model.

**Figure 4:**
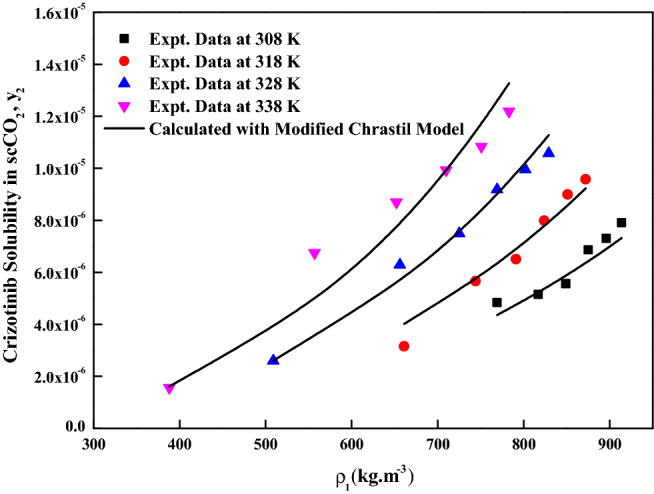
Crizotinib solubility in scCO_2_ versus ρ_1_. Symbols are experimental data points. Solid lines are calculated solubilities with Modified Chrastil model.

**Figure 5:**
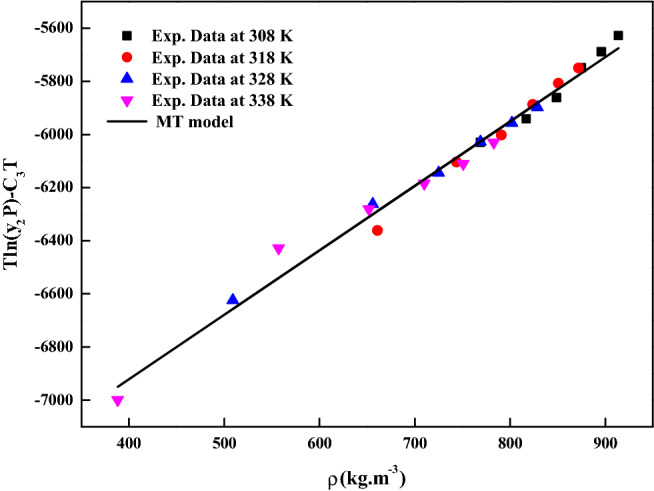
Self-consistency plot based on MT model.

**Figure 6:**
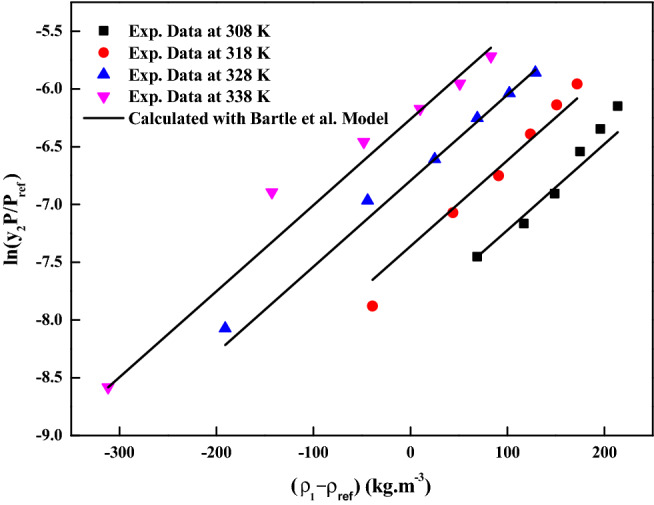
ln(y_2_ P/P_ref_) vs. (ρ_1_–ρ_ref_). Symbols are experimental data points. Solid lines are calculated solubilities with Bartle et al., model.

The reliability of the experimental setup for the solubility measurement was reported in our previous works with naphthalene and alphatocopherol compounds. Crizotinib’s solubilities in scCO_2_ at various conditions are reported in Table 2. Table 2 also indicates scCO_2_ density obtained from NIST database^56^. Table 3 indicates solubility range of some of the anticancer drugs reported in the literature. For the Crizotinib the measured solubilities are observed to range from 0.156 × 10^−5^ to 1.219 × 10^−5^ in mole fraction. Figure 2 indicates solubility isotherms and at 14.5 MPa cross over region is observed. Solubility of pure Crizotinib in scCO_2_ the crossover point (14.5 MPa) is rather unique with respect to temperature. Below 14.5 MPa rise in temperature causes drop in solubility in scCO_2_ phase, while above this point the opposite effect occurs. The following two ways of thermodynamic explanation may be attributed to this phenomenon as at pressures below the crossover pressure the density of the scCO_2_ is more sensitive to temperature changes than at higher pressures. A temperature decrease in this region affects the solubility of the drug in two ways. The vapor pressure of the drug solid decrease while the density of the scCO_2_ (proportional to its solvent power) increase thus the density effect predominates and results in the solubility increase. While on the other hand at pressures above the crossover pressure a temperature increase causes an increase the vapor pressure of the drug while the density of the scCO_2_ decrease thus the vapor pressure effect predominates and the solubility increases^57^. Another way to explain crossover pressure is that at crossover pressure the partial molar configurational enthalpy equals the negative of the sublimation enthalpy^58^. Table 4 indicates all the standard solubility models utilized in this work. Furthermore, it also indicates all the regressed parameters along with AARD%. The correlating ability of these models are shown in Figs. 3, 4, 5 and 6. The solubility data reported in this study is considered self-consistent since all the data is aligned to a single correlation line (Fig. 5). From literature it is clear that Chrastiland modified Chrastil models are useful in calculating thermodynamic properties like heat of reaction and solvation enthalpy of the dissolution process, therefore these models are considered for the correlating the data^31,33^.The total enthalpy of Crizotinib’s dissolution in scCO_2_ is calculated from the Chrastil model parameter A_1_(i.e., $\Delta H_{total} = - A_{1} R$). The sublimation enthalpy of Crizotinibis calculated from Bartle model parameter (i.e.,$\Delta H_{sub} = - B_{4} R$). Sublimation and solvation are the two major steps in dissolution process and the enthalpy of solvation is calculated from the difference between $\Delta H_{total} - \Delta H_{sub}$. A negative sign is attributed to the solvation process. Similar, this approach is used to calculate all these thermodynamic quantities for modified Chrastil and Bartle models combination, thus estimated values are presented in Table 5.

**Table 2:** Solubility of crystalline Crizotinib in scCO_2_ at various temperatures and pressures.

Temperature (K)^a^	Pressure (bar)^a^	Density of ScCO_2_ (kg/m^3^)^56^	y_2_ × 10^5^(Mole fraction)	Experimental standard deviation , S(ȳ) × (10^5^)	S (Equilibrium solubility) (g/L)	Expanded uncertainty of mole fraction (10^5^U)
308	120	769	0.483	0.005	0.038	0.025
	150	817	0.515	0.009	0.043	0.029
	180	849	0.556	0.003	0.048	0.025
	210	875	0.686	0.014	0.061	0.043
	240	896	0.730	0.015	0.067	0.042
	270	914	0.791	0.021	0.074	0.056
318	120	661	0.315	0.011	0.021	0.026
	150	744	0.566	0.015	0.043	0.039
	180	791	0.650	0.011	0.053	0.038
	210	824	0.799	0.016	0.067	0.046
	240	851	0.899	0.020	0.078	0.055
	270	872	0.958	0.041	0.085	0.090
328	120	509	0.260	0.010	0.013	0.022
	150	656	0.629	0.015	0.042	0.042
	180	725	0.749	0.012	0.056	0.043
	210	769	0.918	0.019	0.072	0.056
	240	802	0.995	0.021	0.082	0.061
	270	829	1.057	0.032	0.089	0.079
338	120	388	0.156	0.005	0.006	0.011
	150	557	0.675	0.022	0.038	0.051
	180	652	0.870	0.032	0.058	0.074
	210	710	0.993	0.035	0.072	0.088
	240	751	1.083	0.012	0.083	0.053
	270	783	1.219	0.054	0.098	0.123

The experimentalstandard deviation was obtained by $S(y_{k} ) = \sqrt {\frac{{\sum\limits_{j = 1}^{n} {(y_{j} - \overline{y})^{2} } }}{n - 1}}$. Expanded uncertainty and the relative combined standard uncertainty are (U) = k*u_*combined*_ and *u*_*combined*_*/y* = $\sqrt {\sum\limits_{i = 1}^{N} {(P_{i} {\kern 1pt} u(x_{i} )/x_{i} )^{2} } }$ respectively.

^a^Standard uncertainty u are u(T) =  ± 0.1 K; u(p) =  ± 1 bar. The value of the coverage factor k = 2 was chosen on the basis of the level of confidence of approximately 95 percent.

**Table 3:** Solubility information of the anticancer drugs.

Serial number & name		Solubility range × 10^6^	T(K) and P(MPa) range	Ref.
1	Crizotinibin	0.0156–0.1219	308–338);(12–40)	(present work)
2	Loxoprofen	13.50–1280	(308–338);(12–40)	^9^
3	Tamoxifen	18.80–989.0	(308–338);(12–40)	^10^
4	β-estradiol	0.04–1.12	(308–328);(10.5–22)	^11,18^
5	Imatinib mesylate	0.10–4.41	(308–338);(12–27)	^12^
6	Aprepitant	4.50–76.70	(308–338);(12–33)	^13^
7	Sorafenib tosylate	2.09–12.57	(308–338);(12–27)	^14^
8	Sunitinib malate	5.02–85.62	(308–338);(12–27)	^15^
9	Letrozole	1.60–85.10	(318–348);(12–36)	^16^
10	Azathioprine	2.72–18.27	(308–338);(12–27)	^17^
11	Paclitaxel	1.20–6.20	(308.15–328.15);(10–27.5)	^18^
12	5-fluorouracil	3.80–14.60	(308.15–328.15);(12.5–25)	^11,18^
13	Thymidine	1.20–8	(308.15–328.15);(10–30)	^18^
14	Busulfan	46.90–618	(308–328);(12–40)	^19^
15	Flutamide	4.60–509.3	(308–348);(12.2–35.5)	^20^
16	Gambogic acid	1.63–22.62	(308.15–328.15);(12.2–35.5)	^21^
17	Decitabine	28.4–1070	(308–338);(12–40)	^22^
18	Temozolomide	430–5280	(308–338);(12–40)	^23^
19	Tamusulosin	0.18–10.3	308–338);(12–27)	^24^
20	Pazopanib hydrochloride	1.87–14.25	(308–338);(12–27)	^25^
21	Dasatinib monohydrate	0.45–9.08	(308–338);(12–27)	^26^

**Table 4:** Standard solubility models parameters.

Model	Correlation parameters	AARD%	R^2^	R^2^_adj_
Chrastil model	$\kappa$ = 4.0042; $A_{1}$ = − 20.368; $B_{1}$ = − 3677.8	6.97	0.947	0.939
Modified Chrastil model	$\kappa^{\prime}$ = 4.0048; $A_{2}$ = − 35.727; $B_{2}$ = − 2709	6.95	0.947	0.939
Mendez-Santiago and Teja model	$A_{3}$ = − 7921.4; $B_{3}$ = 2.5303; $C_{3}$ = 9.6455	8.27	0.93	0.919
Bartle et al., model	$A_{4}$ = 11.249; $B_{4}$ = − 5918.3; $C_{4}$ = 7.4427 × 10^−3^	9.18	0.914	0.902

**Table 5:** Calculated thermodynamic properties for Crizotinibin solubility.

Model	Thermodynamic property		
	Total enthalpy, ΔH_total_(kJ/mol)	Enthalpy of sublimation ΔH_sub_(kJ/mol)	Enthalpy of solvation, $\Delta H_{sol}$(kJ/mol)
Chrastil model	30.577^a^		− 18.628^d^
Modified chrastil model	22.522^b^		− 26.683^e^
Bartle et al., model		40.205^c^ (approximate value)	

^d^Obtained as a result of difference between the ΔH_sub_^c^ and ΔH_total_^a^.

^e^Obtained as a result between the ΔH_sub_^c^ and ΔH_total_^b^.

**Figure 7:**
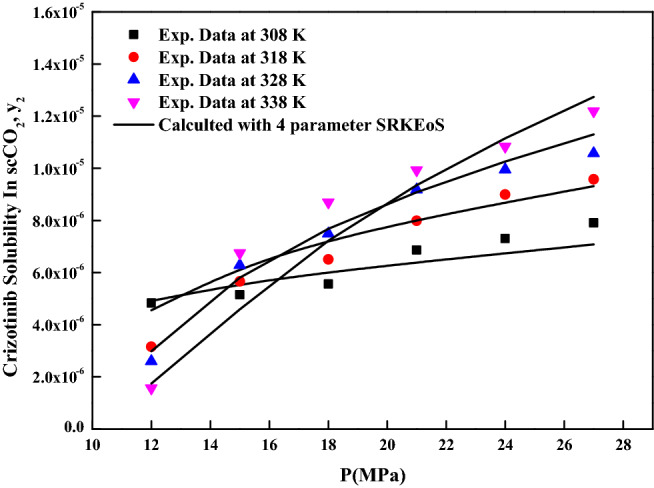
Crizotinib solubility in scCO_2_ vs. P. Symbols are experimental data points. Solid lines are calculated solubilities with SRK EoS + Kwak and Mansoori mixing rules (four parameters model).

The cubic EoS model requires critical properties of Crizotinib and CO_2_ and these are estimated with group contribution methods based on the chemical structure^54,59–61^. Table 6 presents all the estimated critical and physical properties of the drug considered this work. The EoS model regression results are tabulated in Table 7 along with some statistical parameters. From the AARD% it is clear that existing models (RK, SRK EoSs (three parameters) and PR EoS) are poorly correlating the solubility data (Supplementary information Fig. S1, S2, S3). The Crizotinib-scCO_2_ system is highly nonlinear system and to correlate such behavior we may need more adjustable parameter, therefore a new form of solubility model based on Kwak and Mansoori guidelines for SRK EoS is proposed. In the new model, one extra parameter is introduced in the term ‘*a*’ when compared to existing three parameter SRK EoS. Since more parameters are present in the model the regression results will be better in terms of AARD%. Thus, the proposed SRK EoS model is having four parameters. Further the new EoS model is found to correlate the data better than the existing EoS models. From the AARD% it is clear that four parameter SRK EoS correlates the solubility data much better than PR EoS model. Experimental data points and four parameter SRK EoS model predictions are depicted in Fig. 7. Due to poor correlation the RK, PR and three parameter SRK EoSs results are not shown as figures. Overall, four parameter SRK EoS is able to provide satisfactory solubility correlation results. The success of four parameter SRK EoS may be attributed to its number of parameters that constitute the model.

**Table 6:** Critical and physical properties of Crizotinibinand CO_2_^a^.

Substance	T_c_(K)	P_c_(Pa)	$\omega$	V^s^ × 10^−6^ (m^3^/mol)	T(K)			
					P_sub_ (Pa)^f^			
					308	318	328	338
Crizotinib	485.25^b^	14.102^c^	0.4394^d^	32.12^e^	1072	1628	2398	3433
CO_2_	304.18	73.8	0.225					

^a^T_c_:critical temperature; P_c_:critical pressure;*ω* :acentric factor; V^s^:solid molar volume; T:Temperature.

^b^Estimated by Fedors method.

^c^Estimated by Joback and Reed method.

^d^Estimated by Lee-Keslervapour pressure relations (Note: the required normal boiling temperature (at 1.0 atm), T_b_ is estimated with Klincewicz relation, T_c_ = 50.2 − 0.16 M + 1.41 T_b_, where M is molecular weight).

^e^Estimated by Immirzi, A., Perini, B method.

^f^Estimated by Lee-Kesler vapour method.

**Table 7:** Calculated results for the Cubic EoS + Kwak and Mansoori mixing rules.

Model	Correlation parameters	AARD%	R^2^	R^2^_adj_
RK EoS + Kwak and Mansoori mixing rules	$k_{ij}$ = − 0.137	21.7	0.44	0.388
	$l_{ij}$ = − 0.1476			
PR EoS + Kwak and Mansoori mixing rules	$k_{ij}$ = − 0.775	16.2	0.888	0.871
	$l_{ij}$ = − 0.297			
	$m_{ij}$ = 1.810			
SRK EoS + Kwak and Mansoori mixing rules (three parameters)	$k_{ij}$ = 0.367	22.8	0.797	0.767
	$l_{ij}$ = − 0.179			
	$m_{ij}$ = 0.899			
SRK EoS + Kwak and Mansoori mixing rules (four parameters)	$k_{ij}$ = 0.256	8.07	0.921	0.906
	$l_{ij}$ = − 0.236			
	$m_{ij}$ = − 0.293			
	$n_{ij}$ = 0.436

Goodness of the models is quantified using an indicator known as Akaike Information Criterion (AIC) and corrected AIC (AIC_c_)^62–64^. As we know, if the experimental data points are less thanforty, the AIC_c_ is employed. An expression that relates AIC_c_ with AIC, number of data points (N) and number of parameters in the model ($Q$), is

55 $$AIC_{c} = AIC + \frac{{2Q\left( {Q + 1} \right)}}{N - Q - 1}$$

where AIC is $N\;\ln \left( {{{SSE} \mathord{\left/ {\vphantom {{SSE} N}} \right. \kern-\nulldelimiterspace} N}} \right) + 2Q$ and SSE is error sum of squares. According to this indicator, the best model would have least AIC_c_ value. The summary of AIC, AIC_c_, SSE and RMSD values for various models are presented in Table 8. From AIC_c_ values, it is clear that Chrastil and modified Chrastil models are the better models, whereas four parameter SRK EoS model is the best model among EoS models.

**Table 8:** Models and their statistical parameters.

Model	SSE(•10^11^)	RMSE(•10^7^)	N	Q	AIC	AIC_c_
**Standard Solubility Models**						
Chrastil model	1.034	6.564	3	24	− 677.4	− 676.2
Modified Chrastil model	1.033	6.559	3	24	− 677.4	− 676.2
Mendez-Teja model	1.384	7.59	3	24	− 670.4	− 669.2
Bartle et al., model	1.739	8.51	5	24	− 664.9	− 663.7
**Cubic EoS models**						
RK EoS model + Kwak and Mansoori mixing rules	11.90	22.267	2	24	− 620.7	− 620.1
PR EoS model + Kwak and Mansoori mixing rules	3.354	11.822	3	24	− 649.1	− 647.9
SRK EoS model + Kwak and Mansoori mixing rules(Three parameters model)	3.576	12.207	3	24	− 647.6	− 646.4
SRK EoS model + Kwak and Mansoori mixing rules(four parameters model)	1.324	7.428	4	24	− 669.2	− 667.3

## Conclusions

Solubilities of Crizotinib in ScCO_2_ at various conditions are presented at (T = 308, 318, 328 and 338 K) and (P = 12, 15, 18, 21, 24 and 27 MPa), for the first time. The measured solubilities are in the range from 0.156 × 10^−5^ to 1.219 × 10^−5^ in terms of mole fraction. The obtained data was modeled with four standard models and three EoS models combining with Kwak and Mansoori mixing rules. Chrastil and Modified Chrastil models are observed to correlate the data with least AARD% and AIC_c_ values. Among EoS models, four parameter SRK EoS model is able to correlate the data satisfactorily and on par with standard models. Finally, all the standard solubility models considered in this study are able to provide reasonable solubility results.

## Supplementary Information

Supplementary Information.

## Data Availability

The datasets generated and/or analyzed during the current study are not publicly available due to confidential cases and are available from the corresponding author on reasonable request.

## List of symbols

*A*_1_*, B*_1_: Adjustable parameters for Eqs. (6), (7)
*A*_2_*, B*_2_: Adjustable parameters for Eq. (8)
*A*_3_*, B*_3,_* C*_3_: Adjustable parameters for Eq. (9)
*A*_4_*, B*_4,_* C*_4_: Adjustable parameters for Eq. (10)
*AARD%*: Percent of average absolute relative deviation
*AIC*: Akaike information criterion
*AIC*_*c*_: Corrected *AIC*
*C*_*s*_: Concentration of drug inside collection vial in g/L
E1- A14: Apparatus symbols
*H*_sol_: Crizotinib solvation enthalpy in kJ/mol
*H*_vap_: Crizotinib vaporization enthalpy in kJ/mol
*H*_tot_: Total enthalpy of Crizotinib in kJ/mol
k_*ij*_, l_*ij*_*, m*_*ij*_*, n*_*ij*_: Correlation parameters of Eqs. (43) to (46)
$M_{{co_{2} }} ,M_{s}$: Molar mass of CO_2_ and drug in g/mol
$n_{CO2}$: Moles of CO_2_, in mol
$n_{{d{\text{rug}}}}$: Moles of Crizotinibdrug, in mol
*N*: Number of experimental data for Eq. (55)
NIST: National institute of standards and technology
*OF*: Objective function
*P*_*c*_: Critical pressure, in Pa
*Q*: Number of parameters in the model for Eq. (55)
$P_{sub}$: Pure solid sublimation pressure, in MPa
*R*: Universal gas constant, in J/(mol∙K)
*S*: Solubility of Crizotinibin equilibrium state, in g/L
*SSE*: Sum of squares error
scCO_2_: Supercritical carbon dioxide
*T*: Temperature
*T*_c_: Critical temperature
*v*_s_: Molar volume of drug, in m^3^/mol
*V*_1_, *Vs*: Volume of sampling loop and collection vial, respectively, in μL
y_2_, y_i_: Solubility of solute mole fraction
$y_{2,i}^{{{\text{exp}}}} ,y_{2,i}^{{{\text{calc}}}}$: Experimental and calculated solubility points
*Z*: Compressibility factor

## Greek symbols

$\rho$: Density in kg/m^3^
$\rho_{1}$: Mass density of scCO_2_ in kg/m^3^
$\hat{\phi }_{2}^{s}$: Pure substance fugacity coefficient
$\hat{\phi }_{2}^{{scCO_{2} }}$: Fugacity coefficient of solute in fluid phase
*k*: Adjustable parameters for Eqs. (6), (7)
*ω*: Acentric factor
