# Supplementary material for: Experimental solubility and modeling of Crizotinib (anti-cancer medication) in supercritical carbon dioxide
Source: Sci Rep. 2022 Oct 19;12:17494. doi: 10.1038/s41598-022-22366-y (PMC9581934; doi:10.1038/s41598-022-22366-y)
Supplement: Supplementary file 1 — Supplementary Information. [file 41598_2022_22366_MOESM1_ESM.pdf]

Supplementary information

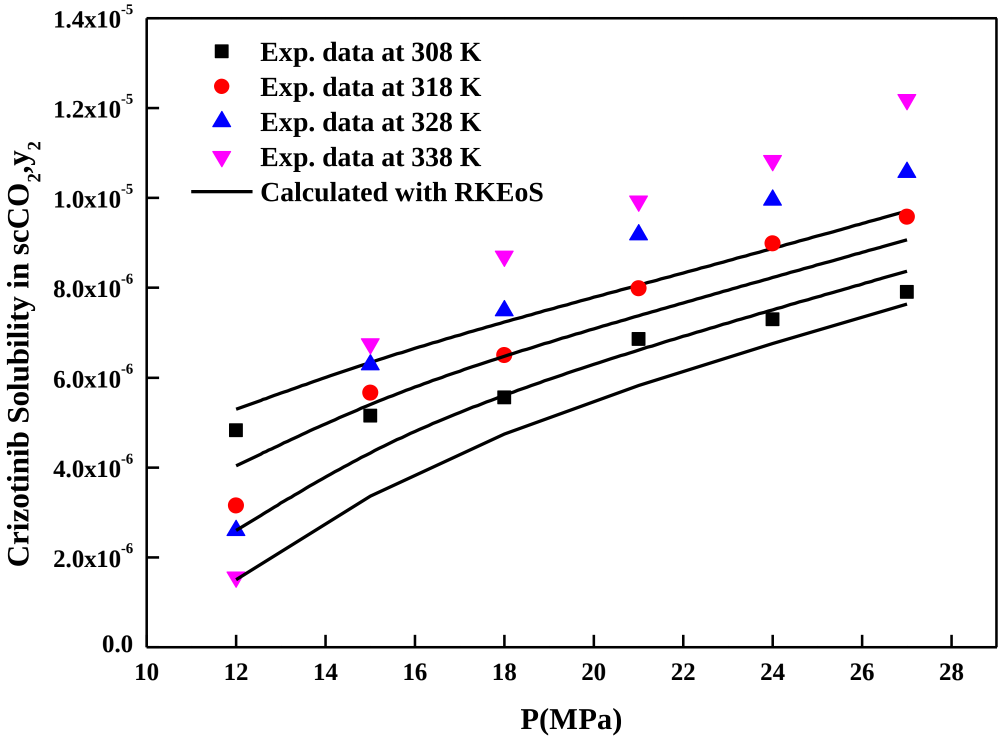

Figure S1. Crizotinib solubility in $scCO_2$ vs. P. Symbols are experimental data points. Solid lines are calculated solubilities with RK EoS+Kwak and Mansoori mixing rules.

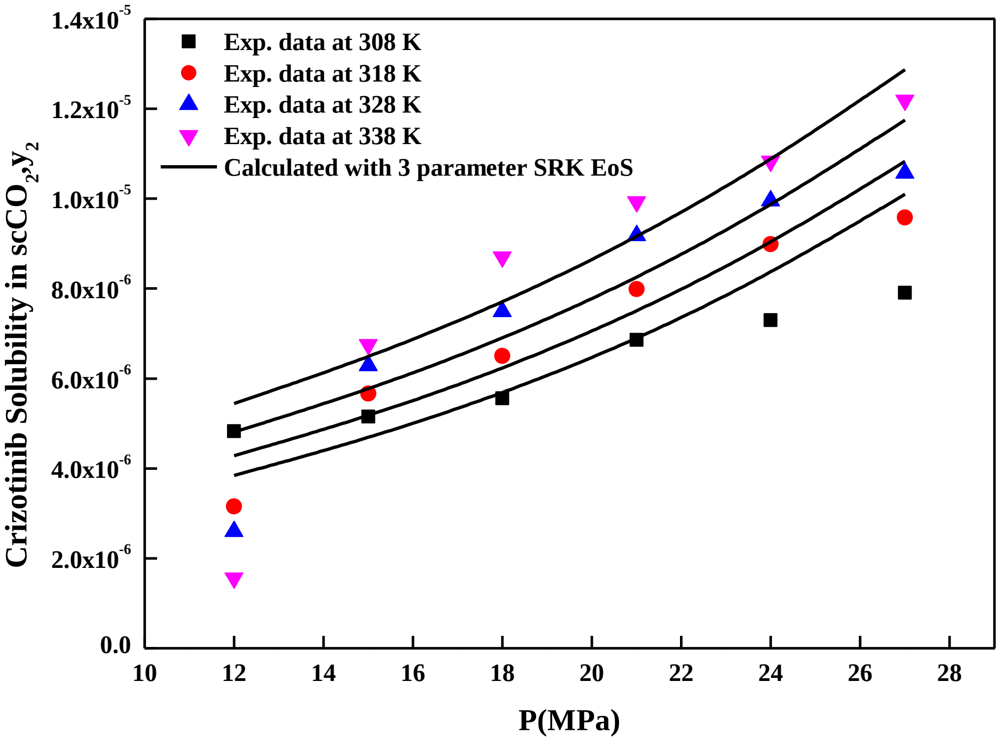

Figure S2. Crizotinib solubility in $scCO_2$ vs. P. Symbols are experimental data points. Solid lines are calculated solubilities with SRK EoS+Kwak and Mansoori mixing rules (three parameters model).

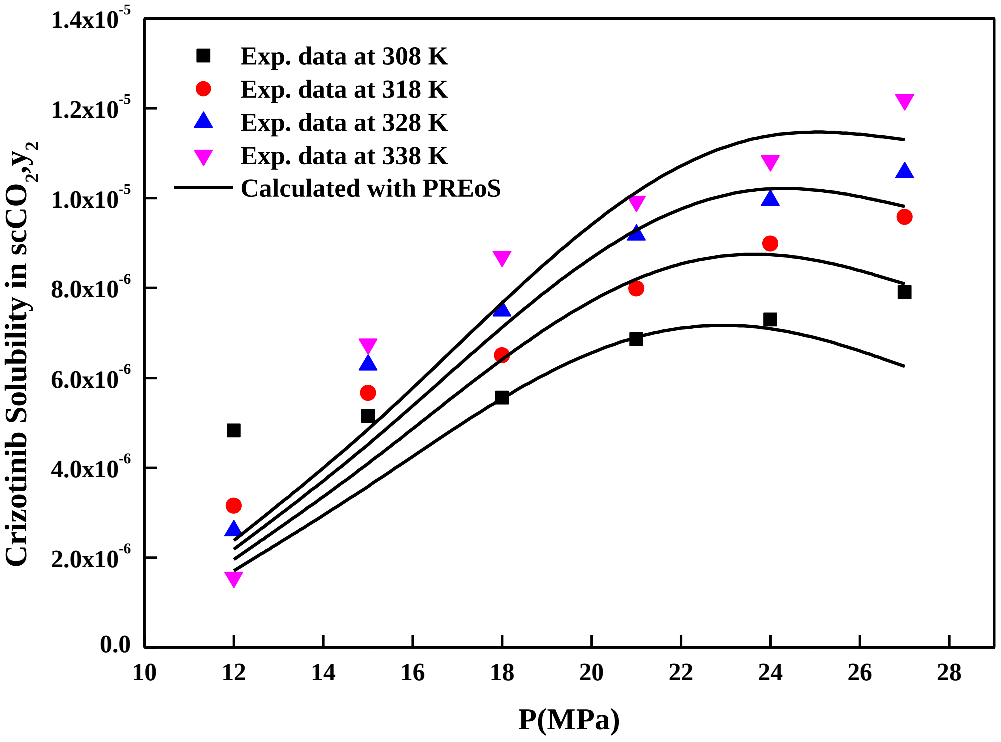

Figure S3. Crizotinib solubility in $scCO_2$ vs. P. Symbols are experimental data points. Solid lines are calculated solubilities with PR EoS+Kwak and Mansoori mixing rules.
